# Extracellular Mitochondrial Components as Emerging Biomarkers and Therapeutic Targets in Human Diseases

**DOI:** 10.7150/ijbs.140360

**Published:** 2026-09-24

**Authors:** Kuan-Hao Tsui, Emmanuel Naveen Raj, Andy P. Tsai, Boyang Wang, Chen-Yueh Wen, Chia-Jung Li

**Affiliations:** 1 Department of Obstetrics and Gynecology, Kaohsiung Veterans General Hospital, Kaohsiung 813, Taiwan.; 2 Institute of Biopharmaceutical Sciences, National Sun Yat-sen University, Kaohsiung 804, Taiwan.; 3 Department of Neurology and Neurological Sciences, Stanford University School of Medicine, Stanford, CA 94305, USA.; 4 Sundial Initiative, Palo Alto, CA, 94304, USA.; 5 Division of Urology, Show Chwan Memorial Hospital, Changhua 500, Taiwan.; 6 Center of General Education, Cheng Shiu University, Kaohsiung 833, Taiwan.; 7 National Museum of Marine Biology & Aquarium, Pingtung 944, Taiwan.

**Keywords:** mitophagy, intercellular transfer, mitochondrial-derived vesicles, lysosomes

## Abstract

Mitochondria serve as the bioenergetic hub of eukaryotic cells while simultaneously acting as a major source of cellular stress and toxicity. Classical mitochondrial quality control (MQC) has long been viewed as a lysosome-dependent process centered on mitophagy. However, aging is accompanied by progressive lysosomal dysfunction, limiting the efficiency of canonical mitochondrial clearance and contributing to mitochondrial accumulation and cellular deterioration. Emerging evidence suggests that under conditions of lysosomal overload and metabolic stress, cells engage alternative quality-control pathways by selectively exporting mitochondrial components into the extracellular environment through mitochondrial-derived vesicles (MDVs) and exophers. In this review, we integrate current findings to propose a unified framework of age-associated mitochondrial disposition and discuss how cells dynamically balance intracellular degradation with extracellular mitochondrial secretion. We further examine the molecular machinery underlying MDV and exopher biogenesis and evaluate their roles in reshaping intercellular metabolic communication. Particular emphasis is placed on their translational relevance in cancer and neurodegenerative disorders, where extracellular mitochondrial trafficking may contribute to disease progression, therapeutic resistance, and tissue remodeling. We also discuss the technical and analytical bottlenecks that currently constrain this field. Finally, we discuss the emerging potential of mitochondrial secretion pathways as diagnostic biomarkers and therapeutic targets for age-associated diseases.

## 1. Introduction: The Evolutionary Paradox and the Limits of Containment

### 1.1. Bioenergetic Benefits and Physiological Constraints of Endosymbiosis

The evolution of the eukaryotic cell is founded upon a singular, transformative endosymbiotic event occurring approximately 1.5 billion years ago, when an archaeal host internalized an 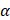-proteobacterium 1-3. This internalization represented a major thermodynamic transition. By integrating a respiratory apparatus capable of oxidative phosphorylation (OXPHOS), the nascent eukaryote bypassed the bioenergetic constraints of glycolysis. The proton motive force generated across the inner mitochondrial membrane (IMM) expanded the cellular energy budget by orders of magnitude, supporting the accumulation of genomic complexity and the subsequent evolution of multicellular life 4, 5. Nevertheless, this metabolic integration imposed inherent physiological trade-offs that continue to constrain modern metazoans. While serving as an indispensable hub for biosynthesis and ATP production, the mitochondrion remains a major source of intracellular stress. Specifically, electron leakage from Complex I and Complex III of the mitochondrial electron transport chain (mETC) inevitably generates reactive oxygen species, primarily superoxide and hydrogen peroxide, directly within the cytoplasmic environment 6.

### 1.2. The Intrinsic Vulnerability of the Power Plant

This close juxtaposition of energy production and reactive oxygen species (ROS) generation creates a precarious local environment within the mitochondrion. Unlike the nuclear genome, which is compartmentalized within the nucleus and protected by an elaborate chromatin architecture composed of histones, the mitochondrial genome is located in the matrix in direct proximity to the primary sites of ROS production 4. As a result, mitochondrial DNA (mtDNA) is uniquely vulnerable to oxidative damage, particularly the formation of oxidized guanine lesions such as 8-oxo-2′-deoxyguanosine (8-oxo-dG), which promote DNA strand breaks and replication errors at rates far exceeding those observed in nuclear DNA 7, 8. In the context of long-lived, post-mitotic cells such as neurons and cardiomyocytes, this progressive accumulation of oxidative damage is particularly detrimental. The progressive accumulation of this damage constitutes the central premise of the mitochondrial theory of aging, which proposes that a gradual decline in respiratory accuracy functions as a circadian rhythms that limits organismal lifespan 9. Importantly, the threat posed by mitochondrial damage extends beyond bioenergetic failure. Owing to the bacterial origin of mitochondria, their molecular components, including N-formyl peptides, cardiolipin, and hypomethylated CpG DNA, retain strong immunostimulatory properties 10, 11. From the perspective of the host innate immune system, a damaged mitochondrion that releases its contents into the cytosol is effectively indistinguishable from an invading bacterial pathogen.

### 1.3. The Threat of Sterile Inflammation

The failure to contain mitochondrial integrity thus instigates a catastrophic signaling cascade. The leakage of mtDNA into the cytosol engages ancient antiviral sensors such as the cGAS-STING pathway, leading to the production of type I interferons (IFNs) and nuclear transcription factor-κB (NF-κB)-driven cytokines 10, 12. Similarly, the binding of oxidized mtDNA to the NLR family pyrin domain containing 3 (NLRP3) inflammasome triggers the proteolytic maturation of Interleukin-1β (IL-1β) and Interleukin-18 (IL-18) 13. While these mechanisms likely evolved to coordinate cellular defense against pathogens, in the context of aging and metabolic disease, they drive “sterile inflammation”—a chronic, low-grade inflammatory state often termed 'inflammaging'. Over decades of life, the chronic leakage of mtDNA and oxidized components acts as a persistent trigger for these immune sensors, establishing the pathological foundation for age-related conditions ranging from neurodegeneration to atherosclerosis and type 2 diabetes 13, 14. Therefore, the sequestration and neutralization of dysfunctional mitochondria is not a housekeeping luxury; it is a biological imperative essential for preventing systemic autoinflammatory collapse.

### 1.4. The Dogma of the Closed Loop

For the past two decades, the conceptual framework for managing this risk has been dominated by a single, monolithic paradigm: intracellular degradation. Since the seminal characterization of autophagy by Christian de Duve and the subsequent mechanistic elucidation by Yoshinori Ohsumi, the field has operated under the assumption that the cell functions as a closed metabolic loop 15. In this model, MQC is synonymous with mitophagy—the selective engulfment of the organelle by autophagosomes and its subsequent hydrolysis within the lysosome 16, 17. This view appeals to the principle of metabolic economy: the cell recycles its own waste to replenish biosynthetic pools of amino acids and lipids. The molecular mapping of the PINK1-Parkin pathway, which tags depolarized mitochondria with ubiquitin chains for lysosomal delivery, seemed to complete this picture, offering an elegant, self-contained solution to the problem of organelle damage 18.

### 1.5. The Paradigm Shift: From Recycling to Ejection

However, scientific dogmas are often defined by the anomalies they fail to explain. A convergence of recent observations across diverse biological disciplines has revealed that the “lysosome-centric” view is insufficient. We now know that when the lysosomal system is overwhelmed by proteotoxic stress, or when the metabolic cost of degradation becomes prohibitive, cells utilize a more radical strategy: the active expulsion of mitochondrial material into the extracellular space 19. Whether observed as MDVs in macrophages, tunneling nanotubes (TNTs) in glioblastoma (GBM), or the dramatic ejection of “exophers” in C. elegans neurons, these phenomena indicate that the cell is an open system capable of outsourcing its waste management 20, 21.

### 1.6. Roadmap of the Review

This review posits that the transition from intracellular degradation to extracellular secretion represents a highly regulated metabolic decision node, rather than a stochastic failure of containment. We will first delineate the molecular machinery that enables the biogenesis of secretory vesicles from the mitochondrial surface. Subsequently, we will define the “signaling logic” that adjudicates between internal recycling and external disposal, focusing on the roles of lysosomal stress and nutrient sensing. Finally, we will explore the profound consequences of this intercellular transfer, arguing that mitochondrial secretion acts as a double-edged sword that can either rescue metabolically compromised tissues or propagate disease pathology across the microenvironment.

## 2. The Internal Defense: Molecular Architectures and the Failure of Mitophagy

### 2.1. The Imperative of Surveillance

The maintenance of a healthy mitochondrial network requires a quality control system of exquisite sensitivity. Given that a single cell may contain hundreds to thousands of mitochondria forming a dynamic reticulum, the removal of dysfunctional units must be spatially precise to avoid the indiscriminate loss of metabolic capacity. Evolution has solved this problem through the development of mitophagy, a specialized form of selective macroautophagy. This process is not a stochastic housekeeping event but a highly regulated surveillance mechanism that adjudicates the fate of the organelle based on its bioenergetic fidelity 22. The molecular machinery governing this process can be broadly categorized into two distinct operational modes: the ubiquitin-dependent pathway, which functions as a general stress response, and the receptor-mediated pathway, which acts as a programmable system responsive to specific developmental or environmental cues.

### 2.2. The Ubiquitin-Dependent Sentinel: The PINK1-Parkin Axis

The primary guardian of mitochondrial integrity in metazoans is the PINK1 and Parkin signaling pathway. This system efficiently translates a bioenergetic cue, namely mitochondrial membrane potential (ΔΨm), into a proteostatic elimination program. Under basal conditions, the serine threonine kinase PINK1, also known as PTEN induced kinase one (PINK1), is imported into the IMM through the translocase of inner mitochondrial membrane 23 (TIM23) complex. Within this compartment, PINK1 undergoes constitutive cleavage by the presenilin associated rhomboid like protease (PARL) and is subsequently degraded by the proteasome 18, 23, 24. This continuous turnover mechanism ensures that cytosolic PINK1 levels remain minimal in healthy and functionally competent mitochondria.

However, upon mitochondrial damage and the consequent dissipation of ΔΨm, PINK1 import is arrested. The kinase accumulates on the OMM in association with the translocase of the outer membrane (TOM) complex, where it dimerizes and auto-phosphorylates 25, 26. The activated PINK1 then executes a critical biochemical operation: it phosphorylates both ubiquitin and the ubiquitin-like domain (ULD) of the cytosolic E3 ligase Parkin at the conserved Serine 65 (Ser65) residue 27, 28. This phosphorylation event induces a profound conformational rearrangement in Parkin, releasing it from an auto-inhibited state and unlocking its E3 ligase activity. Once activated, Parkin catalyzes the rapid assembly of ubiquitin chains on a broad spectrum of OMM proteins, including Mitofusins (Mfn1/2), Voltage-dependent anion channel 1(VDAC1), and Mitochondrial Rho GTPase 1 (Miro1) 29. Importantly, Parkin preferentially assembles K63-linked ubiquitin chains, a topology that serves not as a signal for proteasomal degradation, but as a scaffold for the recruitment of autophagy receptors 30. This process is amplified by a feed-forward loop wherein PINK1 continues to phosphorylate the newly added ubiquitin chains, creating a “phospho-ubiquitin coat” that firmly demarcates the damaged organelle from the healthy network.

### 2.3. The Decoding Machinery: Adaptors and Amplification

The ubiquitin signal is biologically inert until it is decoded by specific selective autophagy receptors/adaptors proteins such as Optineurin (OPTN), NDP52 (also known as CALCOCO2), and TAX1BP1 bind to the phospho-ubiquitin chains via their ubiquitin-binding domains (UBDs) and simultaneously tether the mitochondrion to the nascent autophagosome via their LC3-interacting region (LIR) motifs 31, 32. Crucially, this tethering step involves a second layer of regulation mediated by the kinase TANK-binding kinase 1 (TBK1). TBK1 is recruited to the OMM by adaptor proteins, where it phosphorylates them to enhance their binding affinity for ubiquitin and LC3 33. This “coincidence detection” mechanism ensures that mitophagy is only initiated when a threshold of damage signaling is exceeded, thereby preventing the accidental deletion of functional organelles.

### 2.4. Receptor-Mediated Tuning: Context-Specific Control

Parallel to the PINK1-Parkin system, cells utilize integral membrane receptors to enact mitophagy in response to specific metabolic programs. These receptors, which reside on the OMM, contain LIR motifs that directly engage the autophagic machinery, bypassing the requirement for ubiquitylation. The expression and activity of these receptors are tightly coupled with cellular logic. For instance, BCL-2/adenovirus E1B 19-kDa interacting protein 3 (BNIP3) and BCL-2/adenovirus E1B 19-kDa interacting protein 3-like (BNIP3L/NIX), also known as NIX, are transcriptional targets of Hypoxia-inducible factor 1α (HIF-1α). In hypoxic conditions, their upregulation triggers the removal of mitochondria to prevent ROS generation when oxygen acts as the limiting electron acceptor 34, 35. Similarly, FUN14 domain-containing protein 1 (FUNDC1) responds to the cellular energy status. Under basal conditions, it is phosphorylated and inhibited by Src kinase. Upon mitochondrial uncoupling or exposure to hypoxia, the phosphatase phosphoglycerate mutase 5 (PGAM5) dephosphorylates FUNDC1, thereby activating its LC3 interacting region and promoting the recruitment of LC3 to the mitochondrial surface 36-38. In addition, mitochondrial lipid composition can itself function as a signaling cue. The translocation of cardiolipin, a phospholipid normally restricted to the IMM, to the OMM acts as a direct eat me signal that is recognized by LC3, particularly in contexts of neuronal injury 39.

### 2.5. The Lysosomal Bottleneck: Energetics and Saturation

While the upstream machinery of sequestration is robust, the efficacy of the entire pathway is rate-limited by the terminal step: lysosomal degradation. The lysosome is often mischaracterized as a passive waste bin; in reality, it is a metabolic signaling hub whose function is energetically demanding. The maintenance of the luminal pH (4.5-5.0) required for the activity of over 60 hydrolases (including Cathepsins B, D, and L) relies on the V-ATPase proton pump, which consumes significant ATP 40. The biogenesis of lysosomes is masterfully coordinated by the transcription factor TFEB, also known as transcription factor EB (TFEB), which translocate to the nucleus upon Mechanistic target of rapamycin complex 1 (mTORC1) inhibition or calcium signaling to drive the Coordinated Lysosomal Expression and Regulation (CLEAR) gene network 41, 42.

### 2.6. Digestive Capacity of the Lysosome Determines Mitochondrial Fate

The dependence on lysosomal function represents a critical vulnerability of the cellular quality control system, as lysosomal flux becomes the primary bottleneck under pathological conditions (**Figure 1**). Two major modes of failure can be distinguished that force the cell to divert damaged mitochondria toward alternative disposal pathways. The first, corresponding to Pathway 1 (Competitive Overload) in **Figure 1**, predominates in neurodegenerative disorders such as tauopathies or synucleinopathies, where the accumulation of aggregation-prone proteins competes directly with mitochondria for access to the lysosomal compartment. When the rate of autophagosome formation exceeds the capacity for lysosomal fusion or degradation, the system enters a state of autophagic stress characterized by the cytosolic accumulation of undigested autophagosomes. This congestion obstructs the clearance of ROS producing mitochondria and thereby amplifies cellular toxicity 43-46. The second, corresponding to Pathway 2 (Digestive Impairment) in Figure 1, is a distinct form of lysosomal failure characteristic of metabolic disorders, in which indigestible lipids such as oxidized low-density lipoprotein (OxLDL) or cholesterol crystals accumulate within the lysosome. This lipid burden compromises membrane integrity and induces lysosomal membrane permeabilization, resulting in the leakage of protons and cathepsins into the cytosol. Such disruption not only terminates mitophagic flux but also initiates inflammasome activation 47-52. Within this broader context of digestive failure, in which the intracellular degradation machinery is either overwhelmed or structurally compromised, the adaptive logic of mitochondrial secretion becomes apparent. When the cell can no longer safely degrade its toxic burden, extracellular export emerges as a necessary survival strategy 53-56.

## 3. The Alternative Route: Biogenesis of Secretory Pathways

### 3.1. The Breach of Containment

The canonical view of the cell as a hermetically sealed metabolic unit based on the assumption that organelle turnover is a strictly intracellular process. However, the observation that mitochondrial proteins and genetic material can be consistently detected in the extracellular space, including in blood, cerebrospinal fluid, and the tumor microenvironment, indicates the presence of a regulated breach of this cellular containment 57-60. This phenomenon is not merely a stochastic leak resulting from necrotic membrane rupture. Rather, it represents the operation of specific “secretory programs” that have evolved to bypass the lysosome. We define three distinct, mechanistically conserved pathways that facilitate this export: the budding of MDVs, the formation of TNTs, and the catastrophic ejection via Exophers/Mitoptosis. Each pathway operates at a different scale and kinetic rate, governed by a unique set of molecular logic gates.

### 3.2. Mitochondrial-Derived Vesicles (MDVs): The Molecular Triage

The identification of MDVs by McBride and colleagues constituted a paradigm shift in mitochondrial biology, demonstrating that the organelle is capable of piecemeal degradation without the need for total fission 19. Unlike the “all-or-none” nature of mitophagy, MDV formation is a high-fidelity editing process. It allows the mitochondrion to segregate damaged or oxidized domains while preserving the replicative integrity of the remaining network. The biogenesis of MDVs is mechanistically distinct from the canonical fission machinery used during cell division. While mitochondrial replication relies heavily on the recruitment of the cytosolic guanosine triphosphate (GTPase) Dynamin-related protein 1 (Drp1) to constrict the organelle, the formation of MDVs is predominantly Drp1-independent 61-63. Instead, the process is orchestrated by a specialized protein complex that generates membrane curvature. The sorting nexin 9 (Snx9), typically associated with endocytosis, is recruited to OMM where it interacts with the small GTPase Rab9 (or Rab7 in specific contexts) 61, 62, 64, 65. This complex facilitates the tubulation and scission of vesicles ranging from 70 to 150 nm in diameter.

Crucially, the cargo selection for MDVs is non-random. Quantitative mass spectrometry has revealed that these vesicles are highly enriched for oxidized subunits of the ETC (Complex III subunits like UQCRC1) and specific lipid species. In the classical, oxidative stress-responsive MDV subtype characterized to date, these vesicles are conspicuously devoid of matrix proteins like Pyruvate dehydrogenase complex (PDH) or nucleoids containing mtDNA suggesting a “molecular triage” mechanism: the cell surgically excises the sources of ROS production (the damaged ETC) while retaining the valuable genetic machinery. It should be emphasized, however, that this cargo-exclusion pattern is not universal across all reported MDV subpopulations; alternative MDV subtypes carrying matrix content or nucleoid-associated material have also been described under specific stimuli, indicating that cargo selection is more heterogeneous than a single triage rule would suggest 19, 66, 67. The tagging of this cargo involves the mitochondrial SUMO E3 ligase Mitochondrial anchored protein ligase (MAPL) (also known as MUL1), which sums specific OMM proteins to mark them for vesicular extraction 68, 69. Once scission occurs, MDVs face a bifurcated trafficking fate. While a subset fuses directly with lysosomes, a significant fraction is diverted to the Multivesicular Body (MVB). The entry of MDVs into the MVB is governed by the ESCRT (Endosomal Sorting Complexes Required for Transport) machinery. The ubiquitylated or SUMOylated cargo on the MDV surface recruits ESCRT-I (TSG101) and ESCRT-III (CHMP4B), along with the ATPase Vps4, to drive the invagination of the vesicle into the MVB lumen 19, 70-72. At this stage, the mitochondrial material is topologically transformed into an intraluminal vesicle (ILV). Upon fusion of the MVB with the plasma membrane, a process regulated by Rab27a and Rab27b, ILVs are released into the extracellular space as exosomes 73. This pathway enables the cell to package oxidative stress related cargo and export it to distant recipient cells (**Figure 2**).

### 3.3. Secretory Autophagy: The Amphisome Pathway

Beyond MDVs, the autophagic machinery itself can be repurposed for secretion. Under conditions of lysosomal inhibition or specific inflammatory signaling (IL-1β activation), autophagosomes containing mitochondria do not fuse with lysosomes. Instead, they fuse with the plasma membrane or with MVBs to form hybrid organelles known as amphisomes 74-76. This process, termed “secretory autophagy,” relies on a distinct set of Soluble N-ethylmaleimide sensitive fusion attachment protein receptor (SNARE) proteins, specifically Sec22b, which directs the autophagosome to the cell surface rather than the degradative syntaxin-17 (Stx17) complex 77. This mechanism is particularly relevant in the release of mitochondrial Damage-associated molecular patterns (mtDAMPs). For example, in macrophages stimulated with Lipopolysaccharides (LPS), the release of mtDNA into the cytosol triggers an autophagic capture that results not in digestion, but in the rapid expulsion of the DNA via CD63+ vesicles to alert the immune system 76, 78-80.

### 3.4. TNTs: Direct Intercellular Conduits for Mitochondrial Transfer

While vesicles facilitate diffuse signaling, tunneling nanotubes (TNTs) establish a physical, structural continuity between cells. TNTs are F-actin-based membrane protrusions, with diameters of 50-200 nm and lengths extending up to 100 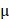
m, that act as transient cytoplasmic bridges 81. The formation of these structures is an active, energy-dependent process upregulated by M-Sec, also known as tumor necrosis factor alpha-induced protein 2 (TNFAIP2), which cooperates with the exocyst complex components RalA and Sec5 to drive actin polymerization at the filopodial tip 82. During intercellular organelle transfer, TNTs serve as dedicated physical conduits. Mitochondria, bound to kinesin motor proteins such as Kinesin family member 5B (Kif5b) via the adaptor protein Miro1, move along internal microtubule tracks within the TNTs to enter the recipient cell 83. This bidirectional transfer is highly relevant in glioblastoma (GBM), where tumor cells extend TNTs to surrounding non-malignant astrocytes. Through these conduits, cancer cells import healthy host mitochondria from the stroma to support their high bioenergetic demands, a directional acquisition that confers resistance to radiation and chemotherapy 84, 85. Conversely, this mechanism can support tissue repair. In models of acute respiratory distress syndrome (ARDS), mesenchymal stem cells (MSCs) utilize TNTs containing Connexin-43 gap junctions to donate mitochondria to damaged alveolar epithelial cells, rescuing them from bioenergetic failure 86, 87.

### 3.5. Mitoptosis and Exophers: The Mechanisms of Extreme Mitochondrial Quality Control

In post-mitotic tissues that cannot rely on cell division to dilute cellular damage, such as the myocardium or the central nervous system, a more radical mechanism exists. Recent work in *C. elegans* neurons has identified exophers—large (approx. 4 μm) membrane-bound extrusions capable of jettisoning a significant fraction of the cell's mitochondrial mass 88. This process, often referred to as mitoptosis in mammalian contexts, is a response to extreme proteostatic stress. Mechanistically, it involves a profound reorganization of the cytoskeleton. The generation of the exopher requires the non-muscle myosin II motor (NMY-2) to generate the contractile force necessary to pinch off such a large volume of cytoplasm 89, 90. Crucially, the mitochondria selected for ejection are functionally compromised, characterized by low membrane potential and high redox stress. In mammalian cardiomyocytes, a similar phenomenon involves the ejection of “respirasomes” within microvesicles, which are subsequently engulfed by resident macrophages to maintain heart homeostasis 91. These diverse mechanisms underscore a fundamental biological principle: mitochondria can function as mobile metabolic entities. Whether packaged in nanometer-scale extracellular vesicles or micrometer-scale exophers, the exported organelle retains bioactive potential. The existence of these elaborate pathways indicates that mitochondrial secretion represents an evolved quality control strategy rather than an accidental cellular byproduct. The pressing question, therefore, concerns the decision logic governing this process. It remains to be determined how the cell integrates metabolic signals to choose between internal lysosomal degradation and external pathways of extracellular secretion.

### 3.6. Technical and Methodological Bottlenecks in the Study of Mitochondrial Secretion

The rapid expansion of interest in extracellular mitochondrial components has outpaced the methodological rigor with which these entities are frequently isolated and characterized, and several bottlenecks currently constrain confident interpretation of the literature. First, distinguishing genuinely secreted, functionally relevant mitochondrial cargo (MDVs, mitoEVs, or exophers released via regulated biogenesis) from cellular debris, apoptotic bodies, or artifacts generated during *in vitro* culture and vesicle isolation remains a substantial technical challenge. Differential centrifugation and standard exosome-enrichment protocols do not reliably separate mitochondria-derived vesicles from co-pelleting apoptotic blebs or free mitochondrial fragments released by incidental cell lysis, and the absence of a single, universally accepted marker for MDVs, as opposed to conventional exosomes marked by tetraspanins such as CD9, CD63, or CD81, compounds this ambiguity 78, 79. Second, the field would benefit substantially from closer adherence to the Minimal Information for Studies of Extracellular Vesicles (MISEV) guidelines, which recommend multi-parametric characterization, including particle counting, protein marker panels, and morphological validation by electron microscopy, rather than reliance on a single readout 78. Applying MISEV-compliant criteria specifically to mitochondrial cargo, for example verifying the presence of outer or inner mitochondrial membrane proteins alongside the absence of markers for other organelles, would help resolve whether a given vesicle preparation represents genuine MDV/mitoEV biogenesis rather than a mixed or contaminated population. Third, evidence for productive intercellular mitochondrial transfer is frequently inferred from the appearance of donor-derived mitochondrial signal within recipient cells, a readout that does not by itself distinguish authentic cytoplasmic fusion and functional integration from simple endocytosis or phagocytic uptake followed by lysosomal degradation of the internalized material. Functional validation, for instance through demonstration of donor mitochondrial genome-encoded respiratory activity in recipient cells, sustained co-localization with the recipient's own mitochondrial network over time, or pharmacological discrimination between phagocytic and fusogenic uptake routes, is necessary to substantiate claims of genuine metabolic rescue or malignant scavenging rather than transient organelle engulfment. We suggest that future studies in this field adopt these more stringent, MISEV-aligned standards to strengthen the evidentiary basis for extracellular mitochondrial biology, particularly as these components are increasingly considered for biomarker and therapeutic applications.

## 4. The Decision Node: Adjudicating Fate

### 4.1. The Logic of Metabolic Governance

The existence of two distinct and functionally divergent pathways for mitochondrial disposition, namely intracellular degradation and extracellular secretion, necessitates the definition of a decision logic that governs mitochondrial trafficking between these fates. This choice is biologically consequential. Mitophagy represents a conservative strategy that preserves cellular homeostasis by recycling amino acids, lipids, and nucleotides to sustain metabolic viability. In contrast, mitochondrial secretion is a dissipative strategy that entails a net loss of biomass and stored energy. The commitment to secretion therefore reflects a state in which the cost of retaining damaged mitochondria exceeds the benefit of recycling them. We propose a hierarchical decision model in which the cell integrates three principal inputs, including lysosomal stress status, nutrient availability, and thresholds of oxidative toxicity. Together, this tripartite evaluation determines whether a damaged mitochondrion is directed toward intracellular degradation or removed from the cell through extracellular export (**Figure 3**). We emphasize that this model is offered as a conceptual synthesis integrating correlative evidence gathered across distinct experimental systems and stress paradigms, rather than a regulatory circuit that has been directly demonstrated within a single, unified experimental setting; it is intended as a heuristic framework to guide future mechanistic testing.

### 4.2. The Lysosomal Stress Sensor: TFEB and the Calcium Switch

The primary determinant of this decision is the functional capacity of the lysosome. As discussed above, when lysosomal flux becomes saturated, the canonical mitophagy pathway is effectively blocked, requiring the cell to sense this impairment and actively reroute mitochondrial trafficking. The central regulator of this adaptive response is the transcription factor TFEB. Under basal conditions, TFEB is phosphorylated by mTORC1 at the lysosomal surface and retained in the cytoplasm through its association with 14-3-3 proteins 92-94. In contrast, lysosomal stress induced by membrane permeabilization, lipid overload, or the accumulation of undegraded cargo triggers the release of calcium from the lysosomal lumen through the Transient receptor potential mucolipin 1 (TRPML1) channel, also known as MCOLN1 95. The resulting localized calcium signaling activates the phosphatase calcineurin, which dephosphorylates TFEB and enables its rapid translocation into the nucleus 96.

Classically, nuclear TFEB is known to drive the expression of the CLEAR network to boost lysosomal biogenesis and autophagy 97, 98** (Figure 4)**. However, recent transcriptomic analyses reveal a duality in TFEB function. In addition to degradative genes, TFEB drives the expression of genes involved in lysosomal exocytosis and vesicle docking, such as LAMP1 and SNARE proteins 74, 99-101. We propose that TFEB may act, in a context-dependent manner, as a “bimodal switch” that couples lysosomal insufficiency to a shift from predominantly degradative toward predominantly secretory gene programs, thereby offering a candidate molecular explanation for why lysosomal storage disorders are frequently characterized by high levels of extracellular vesicles 74, 102-104. It is important to note, however, that TFEB target genes governing lysosomal biogenesis and those governing exocytosis are frequently upregulated concurrently rather than in a strictly sequential, mutually exclusive manner, and the precise molecular determinants that tip this balance toward net secretion have not yet been systematically defined. Candidate mechanisms, none of which have to our knowledge been directly tested in the specific context of mitochondrial disposal, include differences in the amplitude or duration of TRPML1-dependent calcium signaling, with sustained or high-amplitude flux potentially favoring exocytic gene modules; additional post-translational modifications of TFEB beyond calcineurin-mediated dephosphorylation, such as acetylation or SUMOylation; and differential recruitment of coactivators or differential chromatin accessibility at exocytic versus degradative CLEAR-network promoters. We therefore present the bimodal-switch framework explicitly as a testable hypothesis rather than an established mechanism, and this qualification also applies to **Figure 4**.

### 4.3. The Metabolic Checkpoint: mTORC1 vs. AMPK

Superimposed on the lysosomal state is the broader metabolic context of the cell, which is governed by the opposing activities of mTORC1, also known as mechanistic target of rapamycin complex one, and AMP activated protein kinase (AMPK). When cellular energy levels are low, as reflected by a high AMP to ATP ratio, AMPK becomes activated and directly phosphorylates Unc-51 like autophagy activating kinase 1 (ULK1) and the mitochondrial fission factor (MFF) to initiate mitochondrial fission and mitophagy 105-107. Under these energy-depriving conditions, the dominant biological imperative is resource recovery, as the cell cannot afford to lose biomass through secretion and instead prioritizes internal recycling pathways. In contrast, when nutrients are abundant, mTORC1 signaling is activated. Although mTORC1 classically suppresses autophagy through ULK1 inhibition, sustained or hyperactive mTORC1 activity has been associated with enhanced exosome production, particularly in cancer cells 108-110.

In a nutrient rich environment, the energetic cost of discarding damaged mitochondria is relatively low, and mTORC1 driven translational programs further support the production of secretory cargos. We propose that the combination of high nutrient availability and lysosomal stress creates optimal conditions for mitochondrial secretion, allowing the cell to rapidly eliminate dysfunctional mitochondria through extracellular export while simultaneously maintaining mitochondrial homeostasis through ongoing organelle biogenesis, thereby avoiding overload of the intracellular recycling machinery 111-115 (**Table 1**).

### 4.4. The ROS Threshold and Fusion Arrest

The third variable is the toxicity of the cargo itself. The canonical mitophagy process requires the fusion of the autophagosome with the lysosome, a step mediated by the SNARE complex (Stx17, SNAP29, VAMP8) and the Homotypic fusion and protein sorting (HOPS) complex 116-119. However, this fusion machinery is highly sensitive to the local redox environment. Lipid peroxidation products such as 4-hydroxynonenal (4-HNE) are well established to form covalent adducts with cysteine, histidine, and lysine residues on a broad range of proteins, and SNARE proteins have been shown to be susceptible to this type of modification in other cellular contexts 120-123. We therefore propose, as a hypothesis rather than an established mechanism, that severe mitochondrial damage accompanied by excessive ROS production and consequent 4-HNE accumulation could sterically obstruct SNARE-mediated fusion pore formation and thereby impair autophagosome-lysosome fusion. We emphasize that direct experimental demonstration of 4-HNE-SNARE adduction specifically driving the diversion of mitochondrial cargo from mitophagy toward secretion has not, to our knowledge, been reported, and this proposed link should be regarded as plausible but unproven, warranting direct validation, for example through targeted adductomic profiling of SNARE proteins or fusion assays performed under conditions of defined mitochondrial damage. If confirmed, such a physical blockage in the degradative routecould, in principle, prevent the autophagosome (or MDV) from fusing with the lysosome, causing it to accumulate in the cytosol. This accumulation could in turn force the engagement of alternative SNAREs, such as Sec22b, which direct the vesicle toward the plasma membrane for expulsion 124-126. We refer to this hypothetical framework as a “Bypass Model”, in which secretion is envisioned as a failsafe mechanism when internal waste becomes too toxic to handle safely; we present it explicitly as a conceptual hypothesis intended to stimulate future mechanistic investigation rather than as an established regulatory pathway.

### 4.5. The Secretory Phenotype (SASP) Integration

Finally, this decision node is integrated into the broader senescence-associated secretory phenotype (SASP). Cellular senescence, driven by DNA damage or telomere attrition, is characterized by permanent cell cycle arrest accompanied by a marked increase in secretory activity 58, 127-132. Accumulating evidence indicates that mitochondrial dysfunction is a primary driver of this phenotype, a process often referred to as mitochondrial dysfunction-associated senescence (MiDAS) 58, 127-129. Loss of mitochondrial proteostasis activates p38 Mitogen activated protein kinase (MAPK) and nuclear factor kappa B (NF-κB) signaling, which not only induces the expression of inflammatory cytokines but also promotes the biogenesis and release of exosomes containing mitochondrial damage-associated molecular patterns (DAMPs) 129-134. Consequently, the shift from mitophagy to mitochondrial secretion represents a critical transition that commits the cell to a senescent and proinflammatory state 130, 133, 135, 136, thereby fundamentally altering its interactions with the surrounding tissue microenvironment. Therefore, we propose that the secretory expulsion of mitochondria is not merely a transient stress response, but a defining biological hallmark of aged tissues. It represents a tipping point where the age-induced collapse of internal proteostasis (MiDAS) forces the cell into permanent cell cycle arrest, perpetually outsourcing its metabolic waste through SASP. Based on these observations, we propose a hierarchical model governing mitochondrial fate in which healthy lysosomal function and balanced nutrient availability favor basal mitophagy as the most energy-efficient route, metabolic stress and energy depletion activate AMPK to enhance mitophagy for maximal recycling 60, 112, 129, 130, 133, 135-139, and systemic failure resulting from lysosomal saturation or extreme oxidative toxicity diverts mitochondria toward secretion as a survival mechanism. This framework explains why mitochondrial secretion is rarely detected in healthy young tissues but emerges as a defining feature of aging, cancer, and neurodegenerative diseases, conditions in which primary degradative pathways are persistently compromised.

## 5. The Double-Edged Sword: Functional Consequences of Intercellular Transfer

### 5.1. The End of Cell Autonomy

The elucidation of mitochondrial secretion necessitates a fundamental rethinking of classical cell theory. If bioenergetic machinery can be transferred between cells, tissue metabolism can no longer be viewed as the simple sum of autonomous cellular units but must instead be conceptualized as a coordinated and interdependent network. The functional outcome of such mitochondrial transfer is highly context dependent and resembles the concept of a pharmakon, in which the same entity can exert either beneficial or deleterious effects depending on the surrounding metabolic environment and the functional integrity of the transferred organelles. Within this framework, intercellular mitochondrial exchange can manifest in three major pathophysiological modes, including metabolic rescue in stressed tissues, malignant scavenging that supports tumor growth, and inflammatory propagation that amplifies immune activation.

### 5.2. Metabolic Rescue: The Donor's Altruism

In the context of acute tissue injury, the horizontal transfer of healthy mitochondria serves as a potent salvage mechanism. This phenomenon, termed “metabolic rescue,” relies on the capacity of stem cells or stromal support cells to donate functional organelles to distressed tissues. The most definitive evidence comes from models of acute lung injury. MSCs have been shown to transfer mitochondria to alveolar epithelial cells via Connexin-43 gap junctions and TNTs 86, 140-144. Importantly, this is not a passive diffusion but a regulated response to “SOS signals” emitted by the damaged recipient. For instance, damaged cells release ROS which stimulate the upregulation of Miro1 in MSCs, thereby driving the kinesin-mediated transport of mitochondria toward the injury site 145-148. Once internalized, these donor mitochondria do not merely degrade; they fuse with the recipient's network and actively perform oxidative phosphorylation. This exogenous ATP production prevents apoptosis and restores surfactant secretion 5, 148-152. Similarly, in the central nervous system, astrocytes release CD38-dependent mitochondrial particles that are taken up by neurons after ischemic stroke 153-157. This transfer creates a “bioenergetic safety net,” allowing post-mitotic tissues to survive transient metabolic crises that would otherwise be lethal (**Figure 5**).

### 5.3. Unidirectional Mitochondrial Acquisition and Stromal Co-optation in Tumors

In stark contrast to the altruism of stem cells, cancer cells utilize mitochondrial transfer as a mechanism of parasitism. While the Warburg effect characterizes the tumor's reliance on glycolysis, functional mitochondria are still required for pyrimidine synthesis and resistance to apoptosis. In the hypoxic and nutrient-deprived tumor microenvironment, cancer cells hijack mitochondria from surrounding stromal cells to augment their own fitness. This is exemplified in GBM. GBM cells extend ultra-long TNTs into the brain parenchyma, forming physical connections with non-malignant astrocytes. Through these conduits, the tumor cells siphon healthy mitochondria from the astrocytes, effectively draining the host's energy reserves 84, 85. This theft confers a tangible survival advantage. The acquisition of stromal mitochondria leads to a shift toward oxidative metabolism, which significantly enhances resistance to standard chemotherapies like temozolomide 60, 85, 158-160. Furthermore, in hematologic malignancies, acute myeloid leukemia (AML) cells utilize NADPH oxidase 2 (NOX2)-generated superoxide to induce mitochondrial transfer from bone marrow stromal cells, thereby fueling their high proliferative demand 161. Thus, in oncology, mitochondrial secretion pathways are co-opted to drive metabolic flexibility and drug resistance.

### 5.4. Inflammatory Propagation: The Toxic Spreader

The third mode of interaction is perhaps the most insidious. In the aging central nervous system, the secretion of mitochondria serves a local protective role for the donor neuron but has catastrophic consequences for the tissue microenvironment. As established in Section IV, neurons faced with proteostatic failure eject “spheroids” or exophers containing damaged mitochondria and protein aggregates (α-synuclein or β-amyloid) 88, 90, 162, 163. While this ejection may temporarily extend the survival of the individual neuron by reducing its toxic burden, it outsources the problem to the glial network. Microglia and astrocytes, tasked with clearing this debris, become overwhelmed. The phagocytosis of these mitochondrial DAMPs triggers the NLRP3 inflammasome and the release of IL-1β, initiating a state of chronic neuroinflammation 164-168. Worse still, if the secreted vesicles carry “prion-like” protein seeds alongside mitochondrial remnants, they can facilitate the trans-synaptic spreading of pathology to healthy neurons 169-176. This suggests that the progressive nature of Alzheimer's and Parkinson's disease may be driven, in part, by the very mechanisms intended to perform quality control over a lifespan.

## 6. Conclusion and Perspectives: Metabolism as an Open System

The evidence synthesized in this review necessitates a fundamental revision of our understanding of MQC. The traditional view of the cell as a closed and self-contained unit that is solely responsible for degrading its own damaged components is no longer sufficient. Instead, we propose a hierarchical model of mitochondrial disposition in which the cell operates as an open system. Under conditions of homeostasis, damaged mitochondria are efficiently eliminated through mitophagy. However, when this primary internal defense becomes overwhelmed by chronic stress or aging, the cell engages a secondary and external defense mechanism through mitochondrial secretion.

This open system framework resolves the longstanding paradox in aging biology: how long-lived and non-dividing cells manage cumulative mitochondrial damage across decades of life. At the same time, it exposes critical vulnerability, as the decision to expel toxic material converts a cell intrinsic burden into a tissue wide challenge that requires coordinated clearance by neighboring cells. This conceptual shift opens several transformative avenues for future research and therapeutic development. In cancer, tumor cells may depend on the active disposal of damaged mitochondria to evade apoptosis or on the acquisition of functional mitochondria from surrounding cells to sustain metabolic flexibility and resist chemotherapy. Under such conditions, disrupting the molecular routes of mitochondrial transfer, including pathways involving Snx9, Miro1, or Connexin 43, could represent a powerful adjuvant strategy. By blocking mitochondrial trafficking, cancer cells may be forced into irreversible metabolic collapse 143, 158, 159, 177-180.

In contrast, in neurodegenerative disease, the central limitation appears to lie not in secretion itself but in the impaired clearance of expelled mitochondrial debris by glial cells. Therapeutic strategies that enhance the phagocytic capacity of microglia or restore lysosomal competence in astrocytes, for example through activation of TFEB dependent transcriptional programs, may mitigate the chronic inflammation that arises from extracellular mitochondrial accumulation 181-185. Finally, the selective enrichment of oxidatively damaged mitochondrial proteins within extracellular vesicles raises the possibility that mitochondria derived vesicles in circulation could serve as sensitive biomarkers of metabolic stress. Quantification of mitochondrial cargo within plasma exosomes may provide a real time indicator of tissue level lysosomal dysfunction well before overt clinical manifestations become apparent 76, 80, 134, 186-190. Realizing this diagnostic potential will, however, require the field to confront the pre-analytical and analytical challenges outlined in Section 3.6, including standardization of sample collection, processing, and storage conditions, which can substantially alter circulating vesicle yield and composition; adoption of MISEV-compliant, multi-parametric characterization to confirm mitochondrial vesicle identity and purity 78; and harmonization of quantification platforms across laboratories before mitochondrial cargo can be validated as a robust, clinically actionable biomarker.

In conclusion, the mitochondrion should no longer be viewed solely as the powerhouse of the cell but rather as a mobile unit of metabolic information. The transition from intracellular degradation to intercellular exchange represents a highly evolved adaptive response to chronic stress. This open system framework resolves the longstanding paradox in aging biology: how long-lived and non-dividing cells manage cumulative mitochondrial damage across decades of life when internal degradation systems inevitably fail. At the same time, it exposes a critical vulnerability, as the decision to expel toxic material converts a cell-intrinsic burden into a tissue-wide challenge that accelerates the aging of the microenvironment (**Figure 6**).

## Figures and Tables

**Figure 1:**
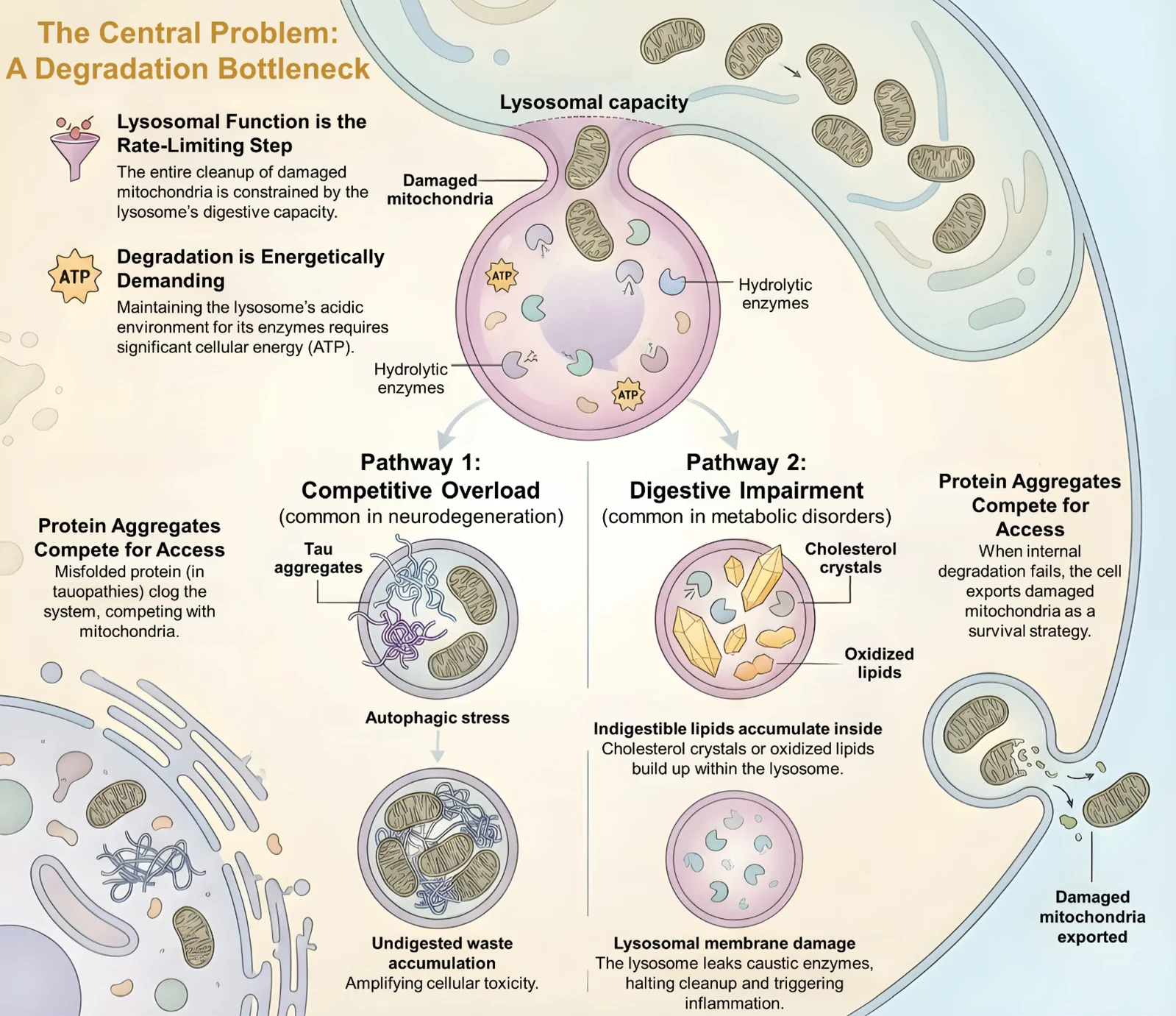
** The Central Problem: A Degradation Bottleneck in Mitochondrial Quality Control (MQC).** The figure shows the limitations of the internal degradation system, where lysosomal function acts as the rate limiting step for clearing damaged mitochondria. This constraint results from the high ATP demand required to maintain the acidic pH for hydrolytic enzyme activity. This degradation bottleneck occurs through two pathological mechanisms that stop mitochondrial clearance. In the first pathway, competitive overload, misfolded protein aggregates compete with damaged mitochondria for lysosomal access, leading to autophagic stress and the accumulation of undigested waste. In the second pathway, digestive impairment, indigestible lipids accumulate inside the lysosome, which causes lysosomal membrane damage (LMD). The resulting leakage of cathepsins and protons stops the cleanup process and triggers inflammation.

**Figure 2:**
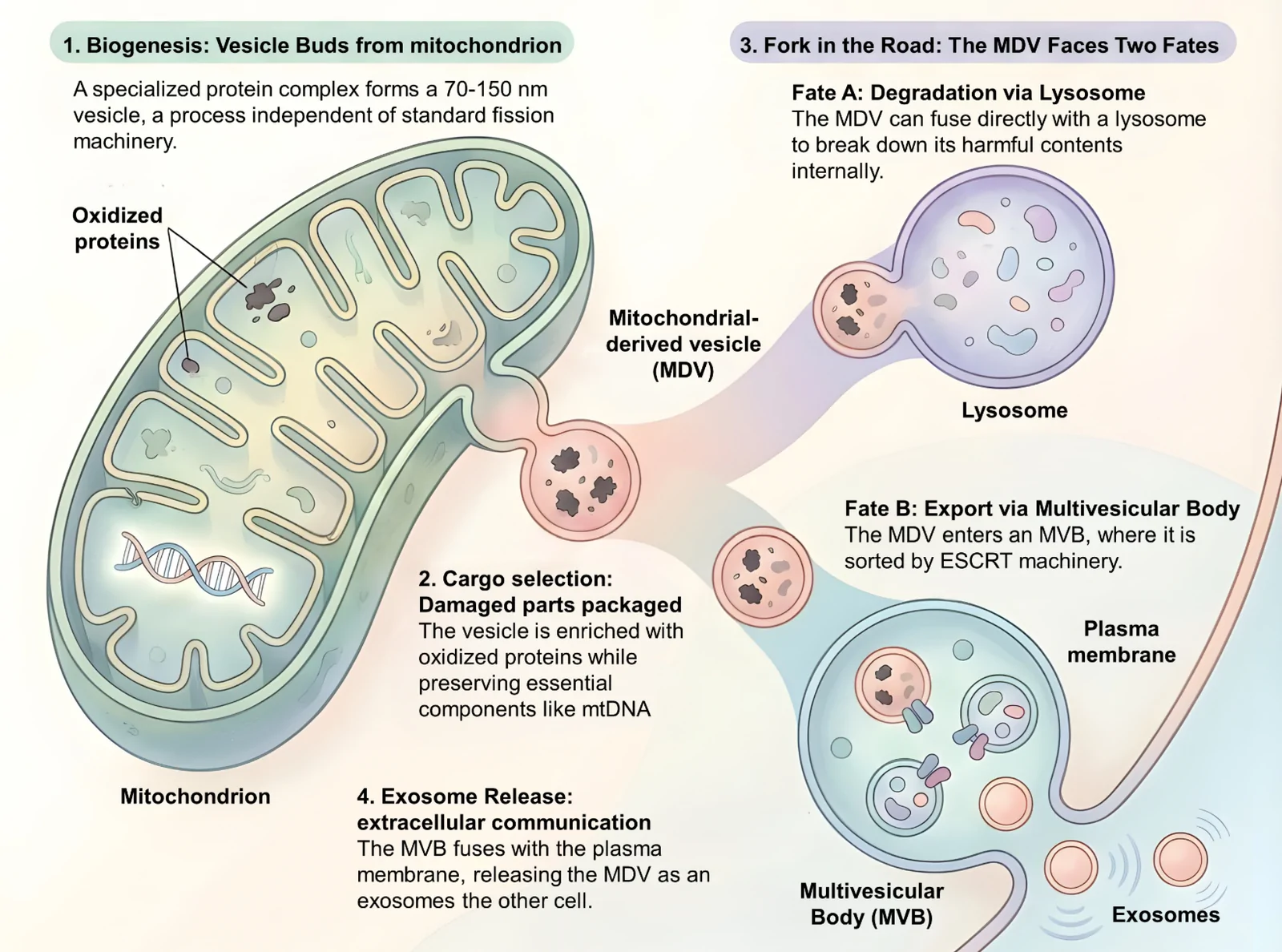
** Biogenesis and Trafficking of Mitochondrial-Derived Vesicles.** The figure shows the dual pathways of MDVs. These 70 to 150 nm vesicles bud from the mitochondrion through a mechanism independent of standard fission machinery. The process begins with cargo selection, where MDVs are enriched with damaged or oxidized proteins while excluding essential components like mtDNA. After formation, MDVs follow one of two routes. The first involves internal degradation, where the MDV fuses directly with the lysosome for breakdown. The second route involves export via the MVB. In this pathway, the MDV enters the MVB and is sorted by the ESCRT machinery. Finally, the MVB fuses with the plasma membrane, releasing the MDV as an exosome into the extracellular space for intercellular communication.

**Figure 3:**
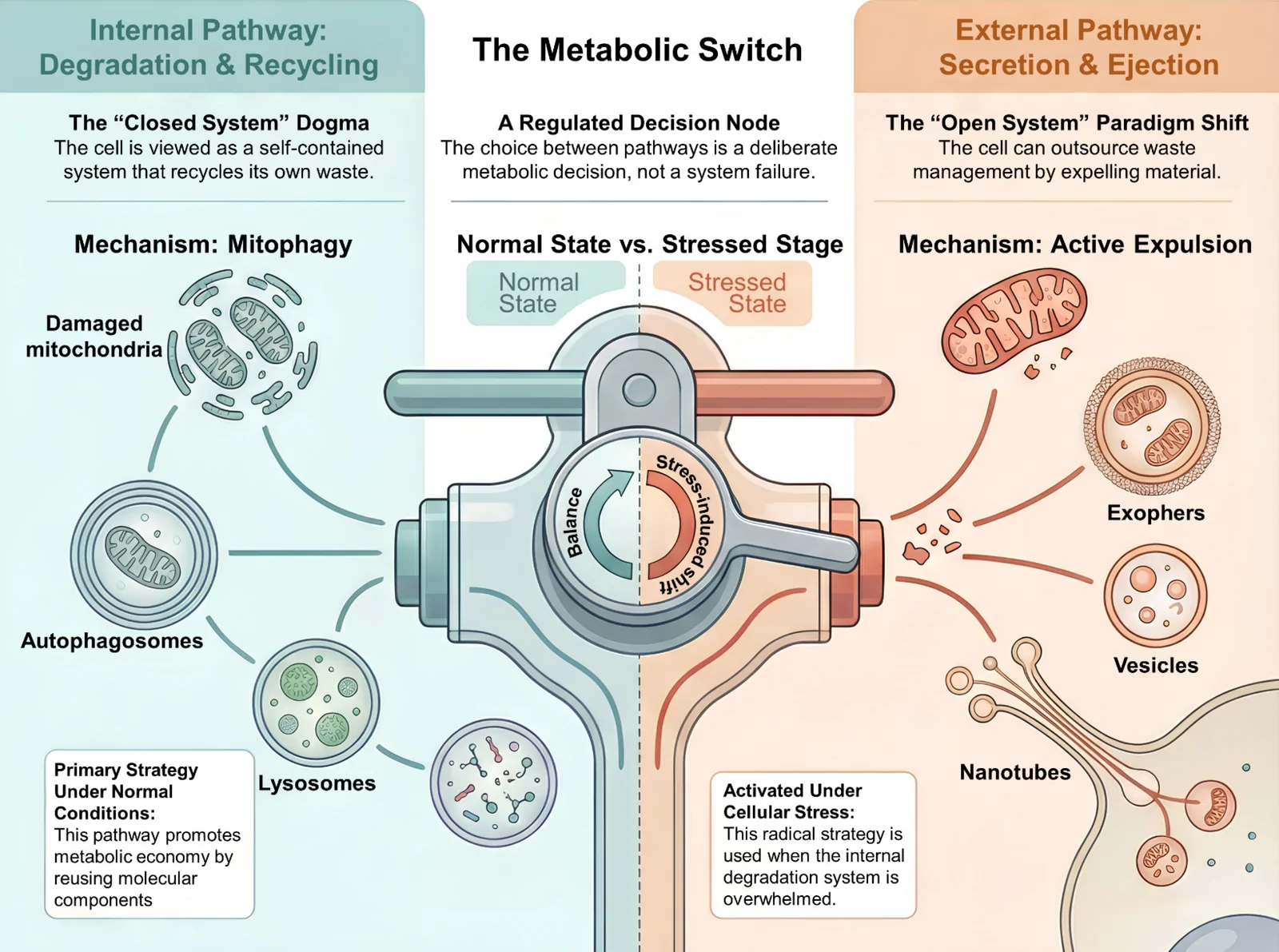
** The Metabolic Switch: Hierarchical Decision Model for Mitochondrial Disposition.** The diagram presents two distinct pathways for MQC regulated by a metabolic switch. Under normal conditions, the cell uses an internal degradation and recycling pathway. In this process, damaged mitochondria are cleared through mitophagy, which involves engulfment by autophagosomes and degradation by lysosomes to maintain metabolic homeostasis and recycle molecular components. When the internal system is overwhelmed or the toxic load is high, the cell shifts to an external secretion and ejection pathway. This mechanism allows the cell to expel material through specialized structures, including vesicles, TNTs, and large cytoplasmic extrusions called exophers. This transition represents a shift in waste management strategy from internal recycling to external disposal. This hierarchical model is proposed as a conceptual framework integrating findings across multiple experimental systems and should be regarded as a hypothesis to guide future mechanistic studies rather than a fully validated regulatory circuit.

**Figure 4:**
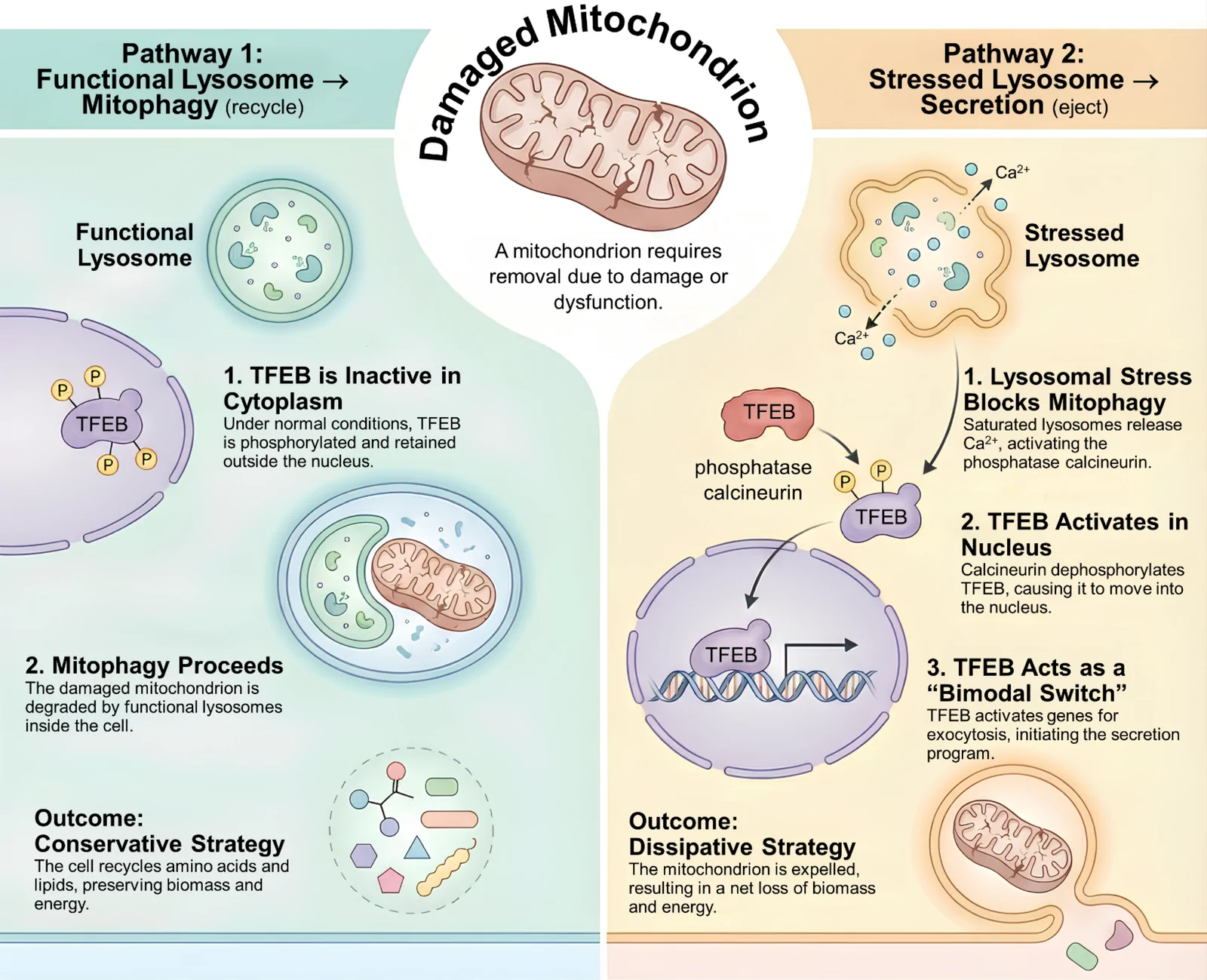
** The TFEB Decision Node: A Bimodal Switch Dictating Mitochondrial Fate.** The figure shows how TFEB regulates whether damaged mitochondria are recycled or ejected. In the first pathway, TFEB remains phosphorylated in the cytoplasm, allowing functional lysosomes to degrade damaged mitochondria through mitophagy. This process recycles cellular biomass and energy. In the second pathway, lysosomal stress caused by toxic cargo triggers the release of calcium ions, which activates the phosphatase calcineurin. Calcineurin dephosphorylates TFEB, leading to its translocation into the nucleus. Once inside, TFEB activates the CLEAR network, which includes genes for lysosomal exocytosis. This results in the secretion of mitochondrial material and a net loss of energy. This bimodal model is a proposed conceptual framework synthesized from the literature rather than a directly demonstrated mechanistic switch; the specific determinants governing the transition from degradative to exocytic gene expression remain to be experimentally established (see Section 4.2).

**Figure 5:**
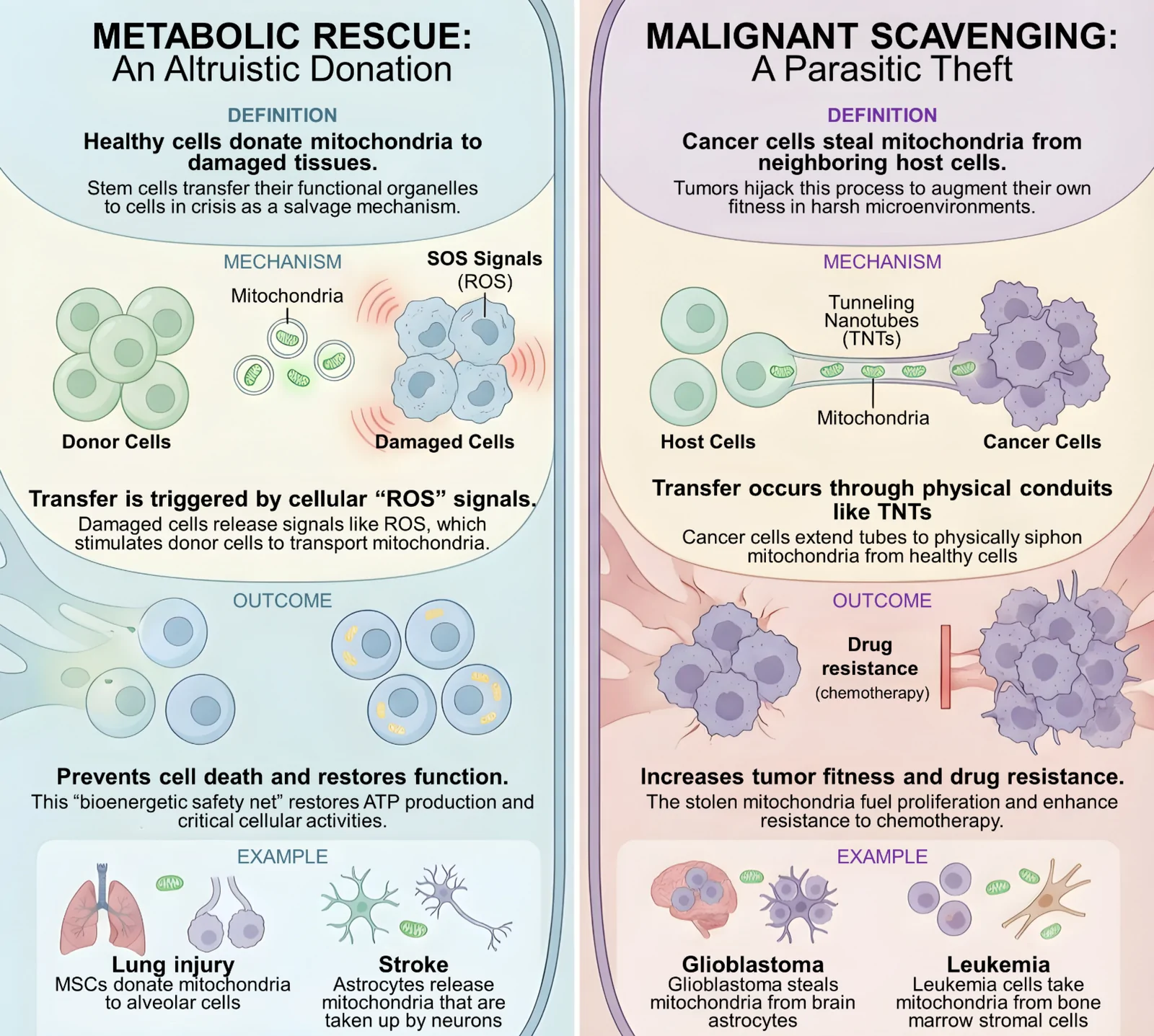
** Intercellular Mitochondrial Transfer: Homeostatic Rescue versus Tumor Co-optation.** The schematic illustrates the dual roles of TNTs in mitochondrial transfer. Left panel: Physiological metabolic rescue, where donor cells (such as stem cells) transfer functional mitochondria to injured cells in response to ROS signaling, restoring bioenergetic function. Right panel: Pathological resource acquisition, where tumor cells utilize long TNTs to import stromal mitochondria, enhancing their metabolic fitness, proliferation, and chemotherapy resistance.

**Figure 6:**
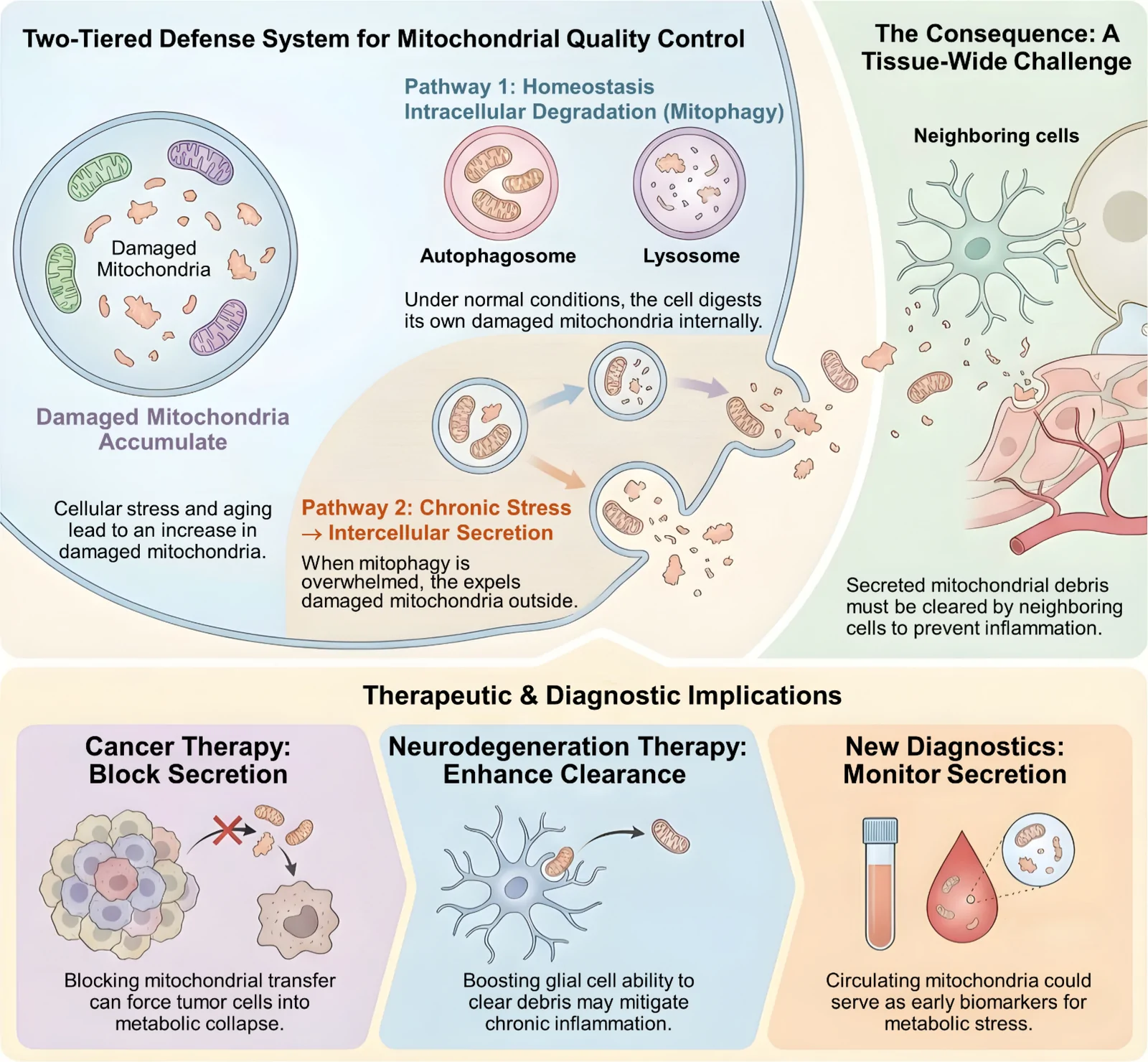
** Two-Tiered Defense System and Therapeutic Implications.** The figure illustrates a two-tiered defense system for MQC, showing the transition from mitophagy to intercellular secretion under chronic stress. This shift occurs when internal degradation capacity is exceeded, requiring neighboring cells to clear the resulting mitochondrial debris. The diagram identifies several translational applications for these pathways. In cancer therapy, blocking mitochondrial secretion may lead to metabolic failure in tumor cells. For neurodegenerative diseases, enhancing the ability of glial cells to clear extracellular mitochondrial material could reduce inflammation. Additionally, circulating mitochondrial components are proposed as biomarkers for the early detection of metabolic stress.

**Table 1:** Comparative Analysis of Intracellular Mitophagy versus Mitochondrial Secretion

Feature	Intracellular Degradation (Mitophagy)	Extracellular Secretion (MDVs / Exophers)
**Primary Biological Logic**	**Recycling (Economy)**	**Disposal (Survival)**
	Recovery of amino acids, lipids, and nucleotides to sustain biosynthesis.	Rapid elimination of toxic material when internal degradation is compromised.
**Triggering Stimulus**	**Nutrient Starvation / Mild Stress**	**Lysosomal Saturation / Proteotoxicity**
	High AMP/ATP ratio; Loss of mitochondrial membrane potential.	Lipid overload; Aggregate accumulation; Extreme ROS.
**Key Signaling Nodes**	**AMPK, PINK1, Parkin**	**TFEB (Nuclear), mTORC1**
	Promotes autophagosome formation.	Promotes vesicle budding and plasma membrane fusion.
**Molecular Machinery**	**Fission-Dependent**	**Fission-Independent (often)**
	Requires Drp1 for fragmentation; LC3 for engulfment.	Requires Snx9/Rab9 for budding; Myosin II for large ejection.
**Terminal Destination**	**Lysosome**	**Extracellular Space / Recipient Cell**
	Acidic hydrolysis.	Exosomes, phagocytosis by neighbors.
**Energetic Cost**	**Net Gain**	**Net Loss**
	Generates substrates for TCA cycle/anabolism.	Consumes ATP for membrane remodeling and transport.
**Physiological Outcome**	**Cellular Autonomy**	**Intercellular Signaling**
	Maintains intrinsic homeostasis.	Metabolic rescue, inflammation, or spreading of pathology.
